# Improving Fatty Acid Availability for Bio-Hydrocarbon Production in *Escherichia coli* by Metabolic Engineering

**DOI:** 10.1371/journal.pone.0078595

**Published:** 2013-10-17

**Authors:** Fengming Lin, Yu Chen, Robert Levine, Kilho Lee, Yingjin Yuan, Xiaoxia Nina Lin

**Affiliations:** 1 Department of Chemical Engineering, University of Michigan, Ann Arbor, Michigan, United States of America; 2 School of Chemical Engineering and Technology, Tianjin University, Tianjin, P.R. China; 3 Department of Biomedical Engineering, University of Michigan, Ann Arbor, Michigan, United States of America; 4 Center for Computational Medicine and Bioinformatics, University of Michigan, Ann Arbor, Michigan, United States of America; University of Groningen, Netherlands

## Abstract

Previous studies have demonstrated the feasibility of producing fatty-acid-derived hydrocarbons in *Escherichia coli*. However, product titers and yields remain low. In this work, we demonstrate new methods for improving fatty acid production by modifying central carbon metabolism and storing fatty acids in triacylglycerol. Based on suggestions from a computational model, we deleted seven genes involved in aerobic respiration, mixed-acid fermentation, and glyoxylate bypass (in the order of *cyoA*, *nuoA*, *ndh*, *adhE*, *dld*, *pta*, and *iclR*) to modify the central carbon metabolic/regulatory networks. These gene deletions led to increased total fatty acids, which were the highest in the mutants containing five or six gene knockouts. Additionally, when two key enzymes in the fatty acid biosynthesis pathway were over-expressed, we observed further increase in strain △*cyoA*△*adhE*△*nuoA*△*ndh*△*pta*△*dld*, leading to 202 mg/g dry cell weight of total fatty acids, ~250% of that in the wild-type strain. Meanwhile, we successfully introduced a triacylglycerol biosynthesis pathway into *E. coli* through heterologous expression of wax ester synthase/acyl-coenzyme:diacylglycerol acyltransferase (WS/DGAT) enzymes. The added pathway improved both the amount and fuel quality of the fatty acids. These new metabolic engineering strategies are providing promising directions for future investigation.

## Introduction

 Fatty-acid-derived hydrocarbons such as alkanes, alkenes, and biodiesel are promising biofuels. Previous research efforts have demonstrated the feasibility of bio-hydrocarbon production in the well-studied model microorganism *Escherichia coli*. For example, Schirmer et al. identified an alkane biosynthesis pathway from cyanobacteria and transferred it into *E. coli*, which led to the production and secretion of C13 to C17 alkanes and alkenes [1]. Meanwhile, Steen et al. demonstrated that *E. coli* could be engineered to produce structurally tailored biodiesel, fatty alcohols, and waxes [2]. However, product titers and yields remain very low, which severely limits the potential of economical and large-scale production of these bio-hydrocarbon fuels. It is believed that part of this inefficiency issue is due to the low availability of their precursors, fatty acids. Therefore, improving the amount, as well as the composition, of fatty acids through metabolic engineering is of great interest.

The past several years have seen increasing efforts in modifying metabolic pathways in *E. coli* to improve fatty acid production, as well summarized in a recent review [3]. Strategies that have been tested and proven effective [2,4-8] can be classified into two broad categories: i) overexpression of enzymes catalyzing key steps in the fatty acid synthesis pathway, including endogenous or heterologous thioesterases (TE), acetyl-CoA carboxylase (ACC), and acyl-CoA ligases (ACL); and ii) deletion of enzymes involved in the β-oxidation pathway that degrades fatty acids, such as acyl-CoA dehydrogenase (FadE), acyl-CoA synthetase (FadD), and a long-chain fatty acid outer membrane transporter (FadL). In one of the latest studies, efforts along this direction led to a titer of 5.1 g/L extracellular fatty acids and a yield of 4.1% (g per g glucose supplied) in a fed-batch culture with online product extraction [8]. In an alternative approach, functional reversal of the β-oxidation cycle has been engineered to produce long chain extracellular fatty acids, which achieved a titer of 7 g/L and a yield of 23% (g per g glucose consumed) [9]. In addition to increasing the quantity of fatty acids produced, work has been done to modify their composition such as chain lengths and saturation levels, which directly affects the quality of the corresponding bio-hydrocarbons. One popular method is to introduce thioesterases from other organisms into *E. coli* to increase medium-chain fatty acids. For example, increases in the levels of medium- and long-chain fatty acids (12:1, 12:0, 14:1, 14:0 and 16:1) were achieved by expressing thioesterase (U31813) from *Cinnamomum camphorum* seeds in an *fadD* knockout *E. coli* strain [7]. Lennen et al. provided another example in which when thioesterase from *Umbellularia californica* was expressed in *E. coli*, the predominant fatty acid chain length was changed dramatically from C16 to C12, with up to 75% of the total fatty acid composition consisting of C12 [4]. Another modification target is the fatty acid saturation level. For instance, the unsaturated fatty acid content in *E. coli* was increased by coexpression of *fabA*, *fabB* and *AtFatA*, leading to a 2.3:1 ratio of unsaturated to saturated fatty acids [10]. 

It should be noted that all the research efforts described above focused on local pathways directly related to fatty acids; rerouting metabolic intermediates in distant central carbon pathways was not considered. The biosynthesis of fatty acids requires metabolic precursors and cofactors, which are provided through the concerted action of a large number of interconnected metabolic pathways. Hence, modifications in distant pathways can potentially improve fatty acid synthesis through redistribution of metabolite precursors or indirect global regulatory effects. For example, the level of malonyl-CoA, a precursor for fatty acids, polyketides and flavonoids, was improved 15-fold through the deletion of *ackA-pta* and *adhE*, together with the overexpression of acetyl-CoA synthetase (Acs) [11]. The recombinant strain was able to produce a 4-fold higher titer of phloroglucinol (an important polyketide), compared to the wild type [11]. Due to the complexity of interconnected metabolic and regulatory networks, accurate computational models can greatly facilitate rational strain design by suggesting genetic manipulations for specific objectives. Many computational algorithms based on metabolic/regulatory networks have been developed for optimization of strains through gene knock-outs, over-expressions and down-regulations, such as OptKnock [12], OptReg [13], OptForce [14], and OptORF [15]. There have been two notable applications of these computational tools that are particularly relevant for fatty acid production. Xu et al. employed OptForce to design an *E. coli* recombinant strain that showed a 4-fold increase of intracellular malonyl-CoA compared to the wild type [16]; Ranganathan et al. also utilized OptForce, which suggested and prioritized genetic manipulations that led to overproduction of fatty acids with chain lengths from C6 to C16 [17].

One key reason for low bio-hydrocarbon yield and efficiency is that free fatty acids, alkanes, and alcohols are toxic to microorganisms and inhibit their growth. Besides elucidating mechanisms of toxicity and engineering tolerant strains [18], another promising approach for increasing fatty acid synthesis is to convert them to triacylglycerol (TAG) and wax ester (WE), the major components of lipid inclusion bodies in oleaginous species. In nature, some gram-positive bacteria are capable of synthesizing substantial amounts of TAG and/or WE, and depositing them as insoluble inclusions in the cytoplasm [19]. It has been reported that TAGs can accumulate up to nearly 76% of the cellular dry matter [20]. These fatty esters provide fatty acyl chains that can be further converted to bio-hydrocarbons [21]. However, compared to *E. coli*, these species capable of TAG and/or WE production exhibit low carbon source flexibilities, require stringent culture conditions, and lack efficient genetic modification tools. Moreover, TAG and WE production with *E. coli* may offer many advantages over traditional production from plant oils or animal fats by circumventing geographical and seasonal limitations [22], allowing a reliable and sustainable supply for bio-hydrocarbon production. In fact, production of WE as biodiesel or as a precursor for other biofuels has been demonstrated in *E. coli* [2,23]. Nevertheless, there has been little investigation on overproduction of TAG in *E. coli*. 

In this work, our goal is to engineer *E. coli* strains to produce fatty acids efficiently and to accumulate fatty acids in the form of TAG, similar to oleaginous species. We carried out a series of genetic modifications on the central carbon metabolism of *E. coli*, suggested by an *in silico* metabolic and regulatory network model, to maximize fatty acid biosynthesis. In addition, we successfully introduced a TAG biosynthesis pathway into *E. coli* to serve as storage for fatty acid carbon chains. 

## Materials and Methods

### *E. coli* strains, plasmids, and culture conditions

Strains and plasmids used in this study are listed in Table 1. All gene manipulations were performed using *E. coli* BL21 Star^TM^ (DE3) (Invitrogen). Gene knockouts were constructed by P1 phage transduction as previously described [24,25]. P1 phage lysates were prepared from Keio single-gene knockout strains [24,25], Δ*cyoA*::kan, Δ*adhE*::kan, Δ*nuoA*::kan, Δ*ndh*::kan, Δ*dld*::kan, Δ*iclR*::kan, and Δ*dgkA*::kan. To knock out simultaneously *nuoA* and *pta*, which are in close proximity on the *E. coli* chromosome, we used strain GNB10760 containing △*nuoA*::FRT △*pta*-*ackA*::kan [26] as the donor strain. The lysates were used to transduce gene knockouts into BL21 Star^TM^ (DE3) and its derivatives. LB agar with 100 μg/mL kanamycin was used as the selective medium. Transductants were purified from residual P1 phage by isolation streaking on LB agar supplemented with 0.8 mM sodium citrate and 100 μg/mL kanamycin, and verified by colony PCR as per published procedures [25]. The FRT-flanked kanamycin resistance gene used for selection was removed by transformation with a temperature-conditional plasmid pCP20 expressing FLP recombinase from a thermoinducible promoter. Multiple knockout mutants were constructed by sequentially implementing the procedure described above and were verified at all disruption sites by PCR at each step when a new mutation was introduced. Plasmids were introduced into BL21 Star^TM^ (DE3) and its derivatives by electroporation transformation. LB agar containing 100 µg/mL ampicillin or 25 µg/mL chloramphenicol was used as the selective medium. 

**Table 1:** Strains and plasmids used in this study.

	**Relevant characteristics**	**Source**
**Strains**		
BL21 Star^TM^ DE3	F^-^ ompT gal dcm me131 lon hsdS_B_ (r_B_m_B_)λ(DE3)	Invitrogen
GNB10760	MG1655, △*nuoA*::FRT △*pta*-*ackA*::FRT-Km-FRT	[26]
△*cyoA*	BL21 Star^TM^ DE3, △*cyoA*::FRT	This study
△*cyoA*△*adhE*	BL21 Star^TM^ DE3, △*cyoA*::FRT △*adhE*::FRT	This study
△*cyoA*△*adhE*△*nuoA*	BL21 Star^TM^ DE3, △*cyoA*::FRT △*adhE*::FRT △*nuoA*::FRT	This study
△*cyoA*△*adhE*△*nuoA*△*ndh*	BL21 Star^TM^ DE3, △*cyoA*::FRT △*adhE*::FRT △*nuoA*::FRT	This study
	△*ndh*::FRT	
△*cyoA*△*adhE*△*nuoA*△*ndh*△*pta*	BL21 Star^TM^ DE3, △*cyoA*::FRT △*adhE*::FRT △*nuoA*::FRT	This study
	△*ndh*::FRT △*pta*-*ackA*::FRT-Km-FRT	
△*cyoA*△*adhE*△*nuoA*△*ndh*△*pta*△*dld*	BL21 Star^TM^ DE3, △*cyoA*::FRT △*adhE*::FRT △*nuoA*::FRT	This study
(aka 6△)	△*ndh*::FRT △*pta*-*ackA*::FRT-Km-FRT △*dld*::FRT	
△*cyoA*△*adhE*△*nuoA*△*ndh*△*pta*△*dld*△*iclR*	BL21 StarTM DE3, △*cyoA*::FRT △*adhE*::FRT △*nuoA*::FRT	This study
(aka 7△)	△*ndh*::FRT △*pta*-*ackA*::FRT-Km-FRT △*dld*::FRT △*iclR*::FRT	
△*dgkA*	BL21 Star^TM^ DE3, △*dgkA*::FRT	This study
SCO0958	BL21 Star^TM^ DE3, pBAD0958	This study
△*dgkA*/SCO0958	BL21 Star^TM^ DE3, △*dgkA*::FRT, pBAD0958	This study
△*dgkA*/WS1	BL21 Star^TM^ DE3, △*dgkA*::FRT, pUCmod-WS1	This study
△*dgkA*/WS2	BL21 Star^TM^ DE3, △*dgkA*::FRT, pUCmod-WS2	This study
△*dgkA*/ATF1	BL21 Star^TM^ DE3, △*dgkA*::FRT, pSK-atf1	This study
△*dgkA*/ATF2	BL21 Star^TM^ DE3, △*dgkA*::FRT, pSK-atf2	This study
6△-△*dgkA*/WS1	BL21 Star^TM^ DE3, △*cyoA*::FRT △*adhE*::FRT △*nuoA*::FRT	This study
	△*ndh*::FRT △*pta*-*ackA*::FRT-Km-FRT △*dld*::FRT △*dgkA*::FRT,	
	pUCmod-WS1	
7△-△*dgkA*/WS1	BL21 Star^TM^ DE3, △*cyoA*::FRT △*adhE*::FRT △*nuoA*::FRT	This study
	△*ndh*::FRT △*pta*-*ackA*::FRT-Km-FRT △*dld*::FRT △*iclR*::FRT	
	△*dgkA*::FRT, pUCmod-WS1	
**Plasmids**		
pMSD8	pFN476 carrying *accBCDA* from *E. coli*	[27]
pMSD15	Carries '*tesA* behind araBAD promoter	[28]
pBAD0958	pBAD33 carrying sco0958 from *Streptomyces coelicolor*	[39]
pUCmod-WS1	pUCmod carrying *ws1* from *Marinobacter hydrocarbonoclasticus*	[40]
	DSM 8798	
PUCmod-WS2	pUCmod carrying *ws2* from *Marinobacter hydrocarbonoclasticus*	[40]
	DSM 8798	
pSK -atf1	pBluescript SK^-^ carrying *atf1* from *Rhodococcus opacus* PD630	[41]
pSK-atf2	pBluescript SK^-^ carrying *atf2* from *Rhodococcus opacus* PD630	[41]

Plasmids pMSD8 and pMSD15 were gifts from Dr. John Cronan. pMSD8 carries *accBCDA* from *E. coli* [27], while pMSD15 contains a native truncated *E. coli* thioesterase '*tesA*, a ‘leaderless’ version of TesA with cytosolic expression [28]. These two plasmids were introduced into the mutant strains by electroporation transformation for the overexpression of *accBCDA* and/or thioesterase.

Unless otherwise stated, M9 minimal medium containing 2% glucose was used for fatty acid analysis of knockout strains. LB Lennox broth, and LB Lennox broth supplemented with different concentrations of glucose and sodium bicarbonate as indicated in the text below were used for characterizing TAG-producing strains. Pre-culture in 50 mL test tubes containing 5 mL of medium was performed overnight at 37°C with 250 rpm shaking. Pre-cultures were diluted 1:100 into 100 mL fresh media in 500-mL conical flasks. Ampicillin (100 µg/mL), chloramphenicol (25 µg/mL), and kanamycin (30 µg/mL) were added as needed. Cells were grown at 37 °C to an optical density at 600nm of 0.6, and then incubated with 0.1 mM isopropyl-β-D-thiogalactoside (IPTG) or 0.2% arabinose. After 24 or 48 hrs of culturing, cell cultures were harvested for analysis.

### *In silico* strain design

A computational framework developed for maximizing biochemical production in *E. coli* [29] was utilized to suggest genetic modifications on the central metabolic and regulatory networks for fatty acid production. We adapted a previously developed regulatory FBA model for central carbon metabolism in *E. coli* [30] and incorporated the fatty acid synthesis pathway. Regulatory relationships involving environmental conditions such as oxygen and substrate availability were represented by logic rules, which determined whether or not related metabolic reactions were active. Then, the *in silico* design framework allowed two types of network modifications - removing reactions and re-activating regulatorily inhibited reactions. It was based on a bi-level optimization formulism. The outer level optimization aimed to maximize the synthesis of a product by identifying reactions for removal/activation, through the use of binary decision variables. The inner level optimization served as a constraint of the outer level problem and simulated the network of reactions dictated by the outer level. It considered mass balance of all metabolites along with other constraints such as reversibility of reactions and determined the flux distribution assuming a cellular objective of maximizing growth. The above bi-level mixed integer linear programming (MILP) problem was transformed into a single-level optimization problem [29] and solved using software Xpress (FICO).

### Analysis of fermentation by-products

Cell growth status was determined by measuring the optical density of the culture at 600nm using a spectrometer. The concentrations of glucose, acetic acid, and lactic acid were measured through HPLC. Culture samples were first filtered through 0.22-μm sterile filters and loaded to an Aminex HPX-87H ion-exchange column (Bio-Rad) operated at 65°C, then eluted with 5 mM H_2_SO_4_ at 0.6 mL/min. Refractive index and UV-vis detectors were utilized. 

### Fatty acid analysis

Duplicates of 50 mL *E. coli* cultures were harvested by centrifugation at 3,200 g x 10 minutes.  Cell pellets were washed with Phosphate Buffered Saline (PBS) and dried at 70°C for 24 hrs. The dry cell pellets were subjected to ethanolysis at 90°C for 2 hrs in 5% HCl in anhydrous ethanol. The resulting Fatty Acid Ethyl Esters (FAEEs) were quantified with an Agilent 6890 GC equipped with a 50 m x 0.2 mm x 0.33 mm HPx5 capillary column, flame ionization detector (FID), and autoinjector. 2 µL FAEE samples were analyzed after split injection (1:10). Helium was used as a carrier gas with a constant flow rate of 1.9 mL/min. The temperatures of the injector and detector were 325 and 350°C, respectively. The following temperature program was applied: 5°C for 3 min, increase 10°C/min to 300°C, then 300°C for 10 min. Quantification was done by utilizing authentic standards.

### Triacylglycerol analysis

Triacylglycerols were identified and quantified as described previously [31]. Dry cell pellets were extracted with 3 mL of n-hexane/isopropanol (HIP, 3:2 v/v). The extraction samples (1mL) were transferred to 1.5 mL GC vials and dried under nitrogen (Visiprep Solid Phase Extraction Vacuum Manifold, Supelco). 10 µL pyridine containing 6,000-8,000 ppm tricaprin as an internal standard and 20 µL *N*-Methyl-*N*-(trimethylsilyl)trifluoroacetamide (MSTFA) were added to the GC vials. The vials were stirred for 1 min and then allowed to react for 30 min at room temperature. Samples were diluted in n-heptane (700 µL), stirred for 1 min, and injected into an Agilent 7890 GC-FID with an ASTM6584 column (15 m x 320 mm x 0.25 um, Agilent J&W). Injection (1.0 µL) was made to a cool-on-column inlet in oven-track mode with an initial oven temperature of 50°C. After a 1-min hold, the temperature was ramped to 180°C at 15°C/min, 230°C at 7°C/min, and 380°C at 30°C/min. Helium was used as carrier gas at a constant flow rate of 3.0 mL/min. The FID detector temperature was 380°C. Nitrogen served as the makeup gas (30 mL/min). Peaks corresponding to TAGs were identified by retention time and quantified based on internal standard calibration of tricaprin.

## Results and Discussion

### Genetic modifications in central carbon metabolism of *E. coli* improves the production of fatty acids

**Figure 1:**
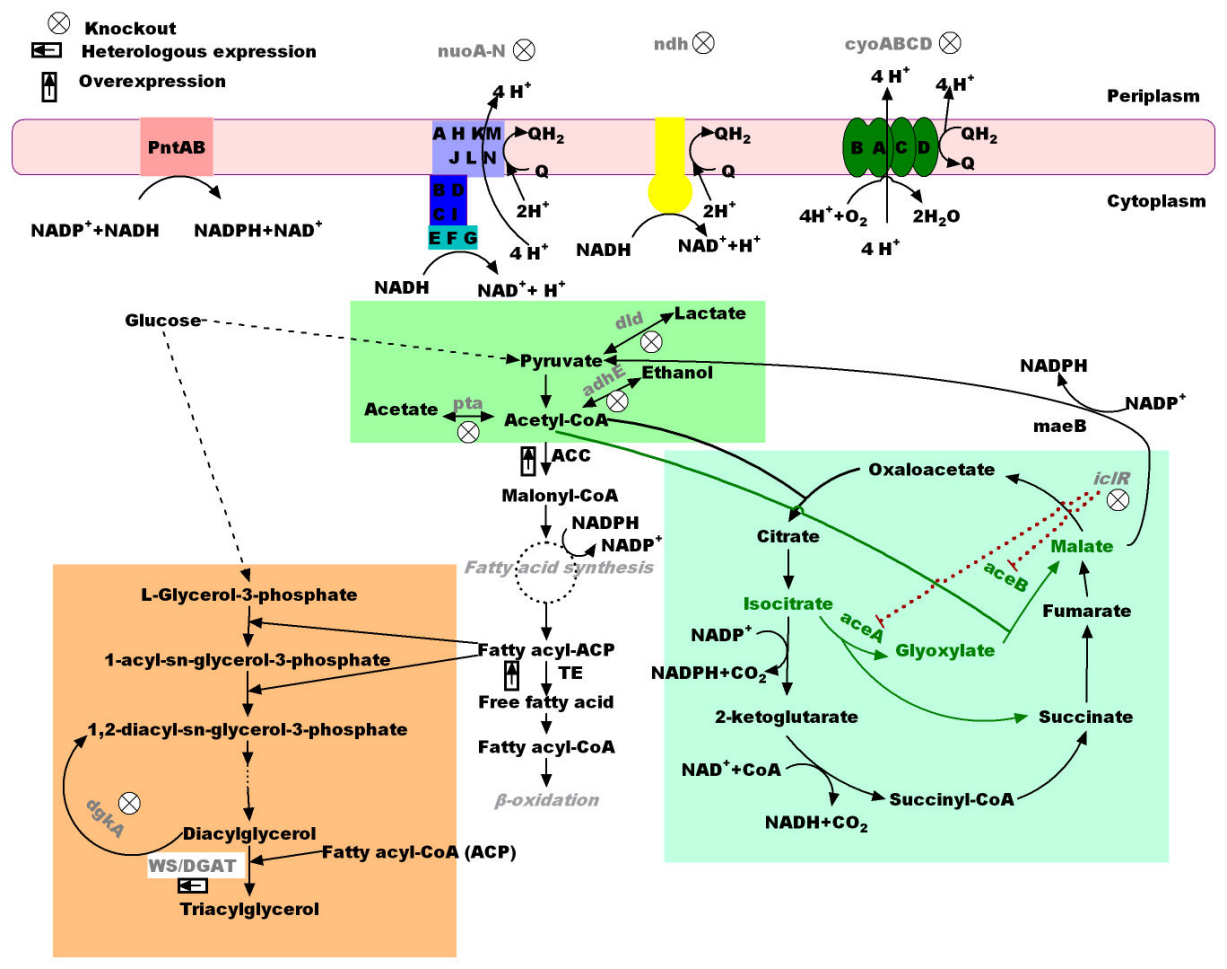
Overview of genetically modified metabolic pathways designed for increasing fatty acid production. Color codes: the orange block contains the triacylglycerol biosynthesis pathway; the green block contains gene manipulations for removal competing pathways in central carbon metabolism; the turquoise block contains the TCA cycle and the glyoxylate bypass pathway, which is shown in green arrows and activated through knockout of the regulatory repressor gene *iclR*.

To create a strain with maximum flux through the fatty acid biosynthesis pathway, we sought to modify the central carbon metabolism in *E. coli*. In this work, we employed an *in silico* modeling and optimization framework that integrates metabolic and regulatory networks to identify targets for genetic manipulations. Similar to OptORF [15], our model allowed two types of network modifications - removing reactions by eliminating enzyme-encoding genes and activating reactions by eliminating regulatory inhibitions [29]. Our metabolic and regulatory network was adapted from a previously developed regulatory FBA model for central carbon metabolism in *E. coli* [30] and incorporated the fatty acid synthesis pathway. Given a specific environment including the carbon source and oxygen condition, this model could be solved to suggest specific network modifications for a desired product [29]. For this work, we were interested in implementing and testing a specific set of network modifications suggested by the above model for synthesizing fatty acids from glucose under aerobic conditions. These network modifications included deletion of six genes, *adhE*, *pta*, *dld*, *cyoA*, *nuoA*, and *ndh*, and activation of the glyoxylate bypass (Figure 1). 

Upon close examination of the corresponding reactions interconnected with others in the network, we hypothesized that this scheme suggested by the model as the optimal design would be promising because it combined two basic strategies: to direct more carbon towards the fatty acid synthesis pathway, by eliminating competing pathways, and to increase the level of cofactor NADPH, which is required by the 3-oxo-acyl-ACP reductase involved in fatty acid biosynthesis [32]. Deletion of genes *adhE*, *pta*, and *dld* belongs to the first strategy, all being common targets in metabolic engineering of *E. coli* for biochemical production (Figure 1). The rest of the suggested manipulations follow the second strategy of increasing NADPH availability, albeit in a more sophisticated manner. Genes *cyoA*, *nuoA* and *ndh* all participate in the aerobic respiratory chain of *E. coli* (Figure 1). *cyoA* encodes subunit II of the cytochrome *bo* terminal oxidase, which catalyzes the two-electron oxidation of ubiquinol into ubiquinone within the inner membrane and the four-electron reduction of molecular oxygen to water. Both *nuoA* and *ndh* are involved in catalyzing the transfer of electrons from NADH to the ubiquinone pool to form ubiquinol. NuoA is part of the NADH:ubiquinone oxidoreductase I (NDH-1); *ndh* encodes the NADH:ubiquinone oxidoreductase II (NDH-2). Removal of these three genes disrupts the oxidation of NADH into NAD^+^. The increase of NADH in the cell leads to its oxidation into NAD^+^ and the coupled reduction of NADP^+^ into NADPH by the membrane-bound pyridine nucleotide transhydrogenase, PntAB [33]. Thus, the level of NADPH might be increased by deletion of genes *cyoA*, *nuoA* and *ndh*. On the other hand, the glyoxylate pathway, catalyzed by two enzymes encoded by *aceA* and *aceB*, bypasses the conversion of isocitrate to succinate in the TCA cycle, during which one NADPH as well as one NADH is generated. The activation of this glyoxylate bypass leads to more malate, which can be converted back to pyruvate and generate one NADPH when catalyzed by *maeB* encoded malate dehydrogenase. Hence activation of the glyoxylate bypass might also lead to more NADPH. This can potentially be achieved by deletion of gene *iclR*, the transcriptional repressor of the glyoxylate bypass operon (*aceBAK*). Expression of the *aceBAK* operon is significantly induced by disruption of *iclR* during growth on glucose [34]. 

**Figure 2:**
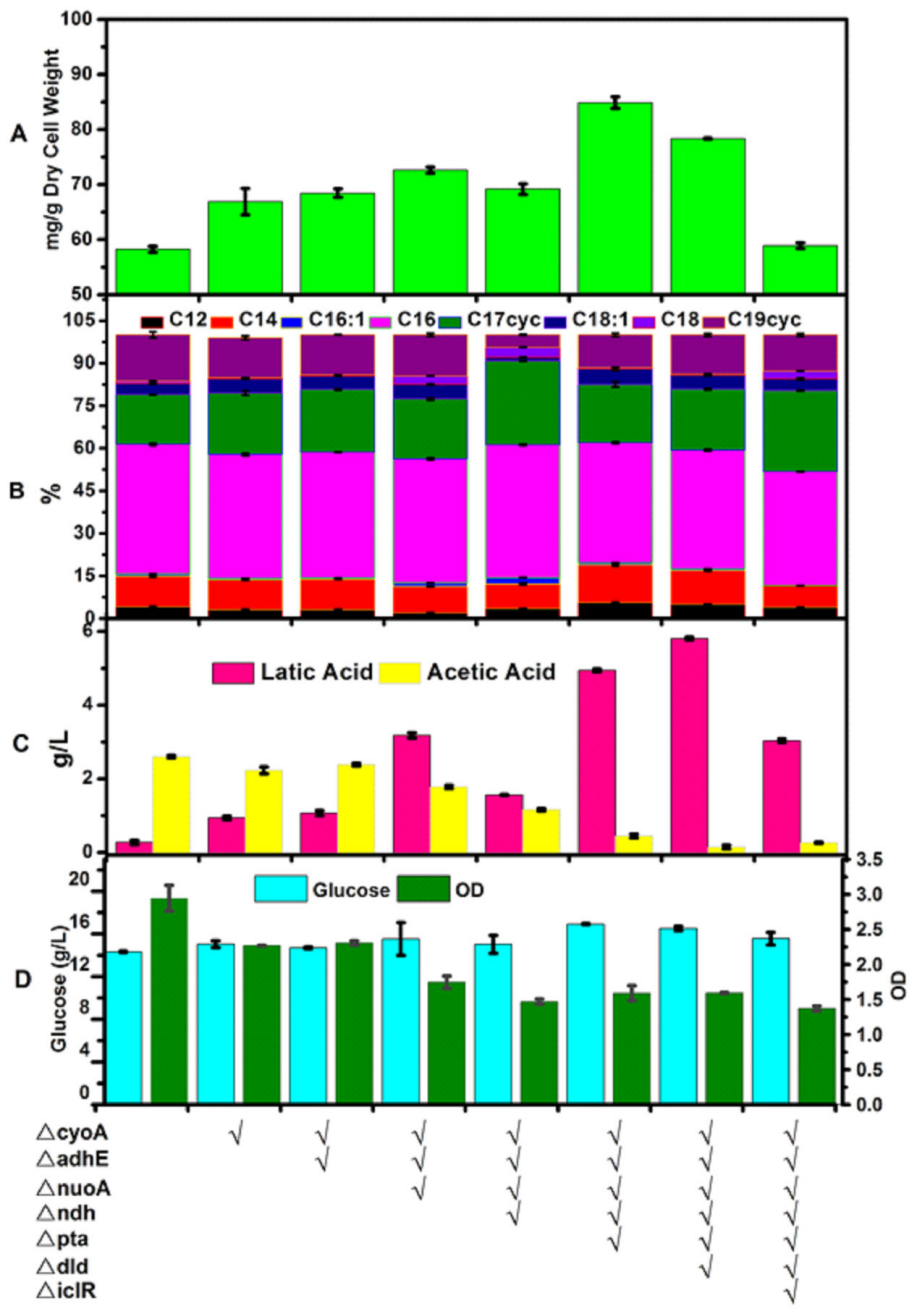
Characterization of *E. coli* strains with genetic modifications in central carbon metabolism. The strains were cultured in M9 minimal medium with 2% glucose for 48 hrs. The total amount of fatty acids (A) and the fatty acid composition (B) were quantified by GC-FID. Other fermentation properties were determined, including the final concentration of by-products lactate and acetate (C), the final cell density (OD), and the final glucose concentration (D). Data presented are averages of two replicate cultures and error bars represent the standard error.

Therefore, we wanted to knock out a total of seven genes and to carry out these knockouts in the order that maximizes the improvement at each step according to *in silico* simulations using flux balance analysis [29]: *cyoA*, *adhE*, *nuoA*, *ndh*, *pta, dld*, and *iclR*. P1 transduction was employed to knock out these targeted genes in the above order. The resulting strains were grown using M9 minimal medium supplemented with 2% glucose. Samples were collected after 24 hrs for quantification of glucose and mixed acids by HPLC, and of fatty acids by GC-FID (Figure 2).

We first created strain Δ*cyoA*. Compared to wild type BL21 Star^TM^ (DE3), the total amount of fatty acids was increased from 58.5 mg/g dry cell weight to 66.9 mg/g dry cell weight in this strain. The final lactic acid concentration was increased from 0.28 g/L to 0.94 g/L, while the final acetic acid concentration was reduced slightly, from 2.6 g/L to 2.2g/L. The final OD decreased from 2.9 to 2.3, whereas the amount of glucose consumed was not affected appreciably. We then deleted *adhE* and created strain △*cyoA*△*adhE*. All the properties described above remain similar to those of strain △*cyoA*. Next we examined strain △*cyoA*△*adhE*△*nuoA*, in which the total amount of fatty acids increased to 72.6 mg/g dry cell weight. The final lactic acid concentration increased substantially to 3.2 g/L; the final acetic acid concentration was reduced slightly to 1.8 g/L. The final OD further decreased to 1.7, whereas glucose consumption remained comparable. Further deletion of *ndh* caused a slight reduction of fatty acid content in strain △*cyoA*△*adhE*△*nuoA*△*ndh*. Despite this unexpected result, we continued with the overall scheme and next removed the phosphate acetyltransferase encoded by *pta*, which is involved in acetate formation from acetyl-CoA. Not surprisingly, the final acetic acid concentration was reduced sharply to 0.45 g/L. This also increased substantially the total amount of fatty acid to 84.9 mg/g dry cell weight, the highest among this set of knockout strains. On the other hand, the final lactic acid concentration was increased to 4.9 g/L. Our second last target was *dld*, which is responsible for lactate biosynthesis. Contrary to model prediction, its deletion reduced the total amount of fatty acids and led to an increase of lactic acid. Finally, we deleted *iclR* in order to activate the glyoxylate pathway. Unexpectedly, the total amount of fatty acids was reduced significantly. After further review of literature related to the glyoxylate pathway, we speculate that there are two possible reasons for this reduced fatty acid production. First, the glyoxylate pathway may not have been effectively activated in our strain (see discussion at the end of this section). Second, even though activation of the glyoxylate bypass pathway could potentially benefit fatty acid synthesis by increasing the NADPH level and preventing carbon loss as CO_2_ in the TCA cycle, this pathway would elevate fatty acid degradation [35] as it is essential for *E. coli* growth on acetate or fatty acids as the sole carbon source [36]. During the strain construction process, in addition to following the above order suggested by our optimization model, we also created several mutants that contained random subsets of these gene knock-outs and did not observe significant improvement of the fatty acid content (Figure S1).

The fatty acid compositions of the above knockout strains were also determined. Fatty acids in *E. coli* exist largely as C12 - C19 molecules, with palmitic acid (C16:0) being the most abundant (Figure 2B). We observed two major changes in the knockout strains. First, deletion of *ndh* from strain △*cyoA*△*adhE*△*nuoA* caused the percentage of C19 cyclopropane fatty acid to decrease substantially. Only 4.4% of the total fatty acid was C19 cyclopropane fatty acid, while the percentage of C19 cyclopropane fatty acid in other strains was in the range of 11%~17%. Second, in strains △*cyoA*△*adhE*△*nuoA*△*ndh*△*pta* and △*cyoA*△*adhE*△*nuoA*△*ndh*△*pta*△*dld*, the portion of medium chain length fatty acids C12 and C14 was higher, which is beneficial for improving the quality of resulted biofuels.

 We have demonstrated that the fatty acid content in *E. coli* can be improved through modification of the central carbon metabolic pathways following suggestions from *in silico* modeling. The total fatty acid content in our best-performing strain △*cyoA*△*adhE*△*nuoA*△*ndh*△*pta* reached 84.9 mg/g dry cell weight, which represented a 45% increase over that in wild type cells, 58.5 mg/g dry cell weight. However, our experimental results did not fully agree with computational predictions and suggest that further optimization is possible. In particular, we have noticed three types of candidates for further genetic modifications. First, there are two other lactate dehydrogenases in *E. coli* encoded by *ldhA* and *lldD*. Knocking them out would completely block the formation of lactate from pyruvate and potentially direct more carbon towards fatty acid biosynthesis. Second, there is another acetate-producing pathway catalyzed by *poxB* encoded pyruvate oxidase. Even though the amount of acetate was quite low after *pta* was deleted in the above study, it is likely that further knockout of *poxB* would lead to complete elimination of this by-product. Last, it has been suggested that derepression of the glyoxylate bypass by *iclR* deletion alone cannot draw isocitrate from the TCA cycle to the glyoxylate bypass because enzyme IcdA has a stronger affinity to isocitrate than enzymes AceA and AceB [37]. Hence, to fully activate the glyoxylate bypass, *icdA* will need to be knocked out in addition to *iclR*. These genetic modifications will be explored in our future study.

### Combining modifications of central carbon metabolism and of fatty acid biosynthesis pathway further improves fatty acid production

**Figure 3:**
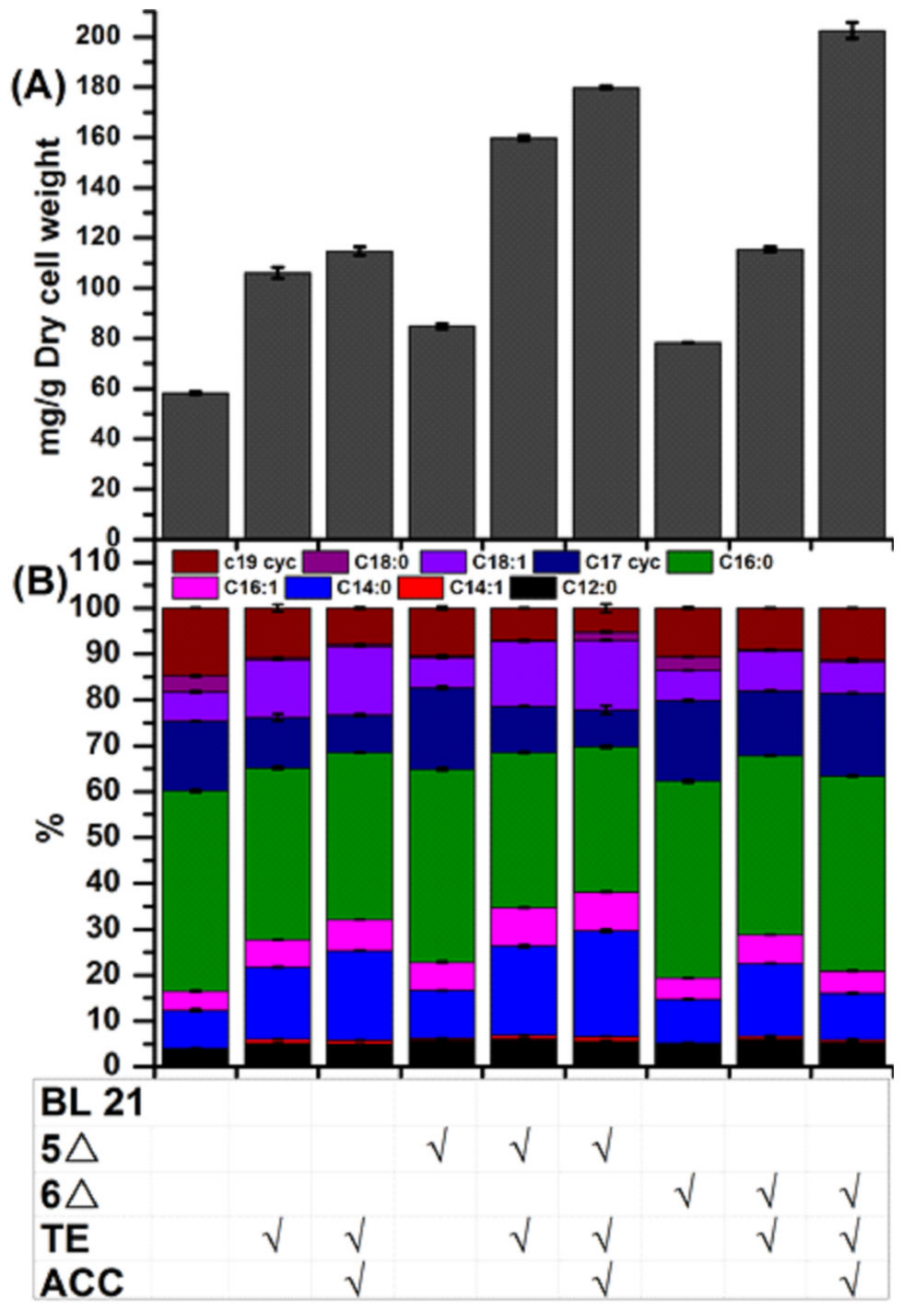
The total fatty acid content in *E. coli* strains with genetic modifications in both central carbon metabolism and fatty acid biosynthesis pathway (A) and the corresponding fatty acid composition (B). The strains were cultured in M9 minimal medium with 2% glucose for 48 hrs. BL 21: BL 21 Star^TM^ DE3; 5△: △*cyoA*△*adhE*△*nuoA*△*ndh*△*pta*; 6△: △*cyoA*△*adhE*△*nuoA*△*ndh*△*pta*△*dld*; TE: a leaderless version of TesA that is targeted to the cytosol; ACC: Acetyl-CoA Carboxylase. Data presented are averages of two replicate cultures and error bars represent the standard error.

With increased availability of metabolic precursor (i.e. acetyl-CoA) and cofactor (i.e. NADPH) from modifications of central carbon metabolism, the capacity of the fatty acid biosynthesis pathway may become a bottleneck. Therefore, combining the rewiring of central metabolic/regulatory networks and modifications of local pathways, as explored in previous studies [2,4,7], could potentially lead to further improvement of fatty acid production. We tested this idea with two genetic manipulations frequently applied to the fatty acid pathway: overexpression of acetyl-CoA carboxylase (ACC), which catalyzes the formation of malonyl-CoA from acetyl-CoA and was found to be a rate-limiting step [27]; and overexpression of thioesterase (TE), which catalyzes the conversion of fatty acyl-ACPs to free fatty acids, hence alleviating feedback inhibition of enzymes involved in fatty acid biosynthesis by long-chain fatty acyl-ACPs [38]. Specifically, we overexpressed the endogenous TE and ACC in the knockout strains △*cyoA*△*adhE*△*nuoA*△*ndh*△*pta* and △*cyoA*△*adhE*△*nuoA*△*ndh*△*pta*△*dld*, termed 5△ and 6△, respectively. As shown in Figure 3A, when TE was overexpressed in the base strain BL 21 and strains 5△ and 6△, the total fatty acid content increased in all three strains. Next, coexpression of TE and ACC further boosted the total fatty acids in each strain. Interestingly, the improvement in strains 5△ and 6△ were substantially larger than that in the base strain. In particular, the total fatty acid content in strain 6△ with overexpression of TE and ACC increased remarkably to 202 mg/g dry cell weight, which is ~250% of that in the wild type. This significant improvement in strain 6△ suggests that both ACC and TE, which catalyze the first and last steps, respectively, of the fatty acid synthesis pathway from acetyl-CoA to free fatty acids, become rate-limiting only after the major competing pathways are removed. These results clearly demonstrate that modifying central carbon metabolism and the local pathway have a synergistic effect on increasing fatty acid production. We also quantified the fatty acid composition in each strain (Figure 3B) and observed that when TE and ACC were overexpressed in knockout strain 5△, the fraction of medium chain length (C12 and C14) and unsaturated (C16:1 and C18:1) fatty acids increased.

### Heterologous expression of WS/DGAT enzymes enables production of triacylglycerol in *E. coli*

**Figure 4:**
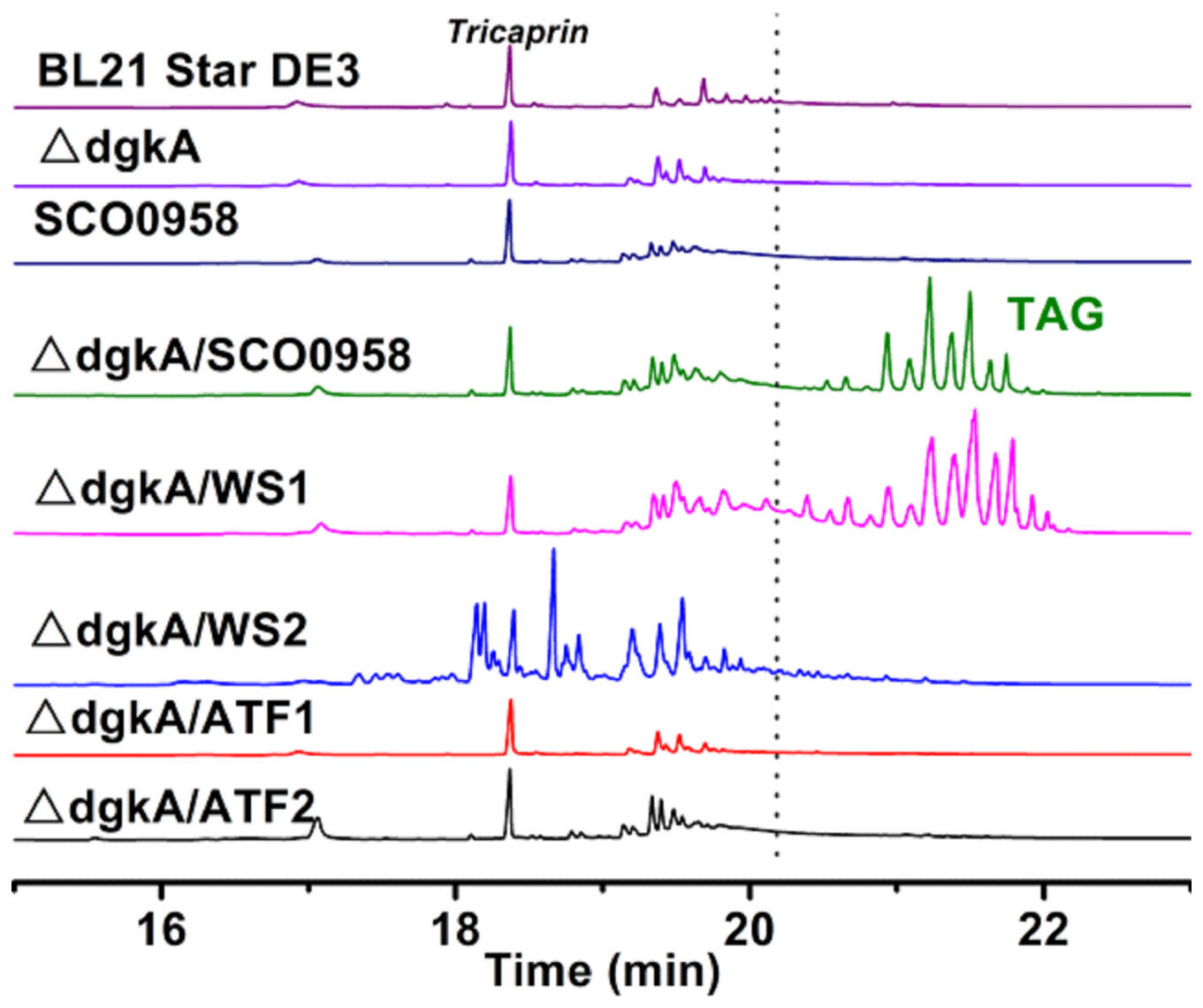
GC chromatograms of triacylglycerol from strains BL 21 Star^TM^ DE3, △*dgkA*, SCO0958, △*dgkA*/SCO0958, △*dgkA*/ATF1, △*dgkA*/ATF2, △*dgkA*/WS1 and △*dgkA*/WS2. All strains were cultured in LB at 30 °C. The location of the TAG peaks is indicated in the figure. Tricarpin is used as an internal standard.

To enable *E. coli* to produce tryacylglycerol, we overexpressed a wax ester synthase/acyl-coenzyme: diacylglycerol acyltransferase (WS/DGAT) enzyme from *Streptomyces coelicolor* (Sco0958) in BL21 Star^TM^ DE3. No TAG production was observed in the recombinant strain (Figure 4). We then knocked out a diacylglycerol kinase (*dgkA*), based on a previous report that Sco0958 possessed DGAT activity and its overexpression in a *dgkA* knockout mutant of *E. coli* led to the detection of TAG by thin layer chromatogram (TLC) [39]. In *E. coli*, *dgkA* is involved in the recycle of diacylglycerol to phosphatic acid. Diacylglycerol is produced as a by-product in Membrane-Derived Oligosaccharide (MDO) biosynthesis and serves as the immediate precursor of TAG (Figure 1). The resulting strain △*dgkA* /SCO0958 showed TAG peaks on GC chromatograph (Figure 4), demonstrating the production of triacylglycerol TAG in *E. coli*. 

 To identify the most active WS/DGAT enzyme in *E. coli*, four other WS/DGAT enzymes from different oleaginous bacteria (Table 1) were introduced into strain △*dgkA*. First, WS1 and WS2 from *Marinobacter hydrocarbonoclasticus* DSM 8798 were tested. TAGs were observed in strain △*dgkA*/WS1, but not in strain △*dgkA*/WS2. This is consistent with a previous *in vitro* study where TAG product was only detected for WS1 when oleoyl-CoA and dipalmitoyl-glycerol were used as substrates [40]. Interestingly, strain △*dgkA*/WS2 showed a drastically different GC chromatogram with new peaks (Figure 4), which indicated that new compounds were produced in *E. coli*. WS2 has been reported to have very high WS activity [40], suggesting that these new compounds are probably wax esters. Further study will be needed to determine the exact identify of these compounds. We also tested two WS/DGAT enzymes from *Rhodococcus opacus* PD630, ATF1 and ATF2 [41]. Neither strain △*dgkA*/ATF1 nor strain △*dgkA*/ATF2 produced TAGs (Figure 4). It was previously found that crude protein extracts of recombinant *E. coli* strains containing ATF1 and ATF2 individually exhibited low WS and DGAT activities, when [1-^14^C] palmitoyl-CoA and 1-hexadecanol were used as substrates for testing WS activity, and [1-^14^C] palmitoyl-CoA and 1,2-dipalmitoylglycerol for DGAT [41]. It is worth noting that *Marinobacter* is a genus of proteobacteria, which also includes *Escherichia*, while *Rhodococcus* is a genus of aerobic, nonsporulating, nonmotile gram-positive bacteria. Hence, *Marinobacter hydrocarbonoclasticus* DSM 8798 is a much closer relative of *E. coli* than *Rhodococcus opacus* PD630, which might account for the high enzymatic activity of WS1 and WS2, in contrast to the dysfunction of ATF1 and ATF2 in *E. coli*. 

**Figure 5:**
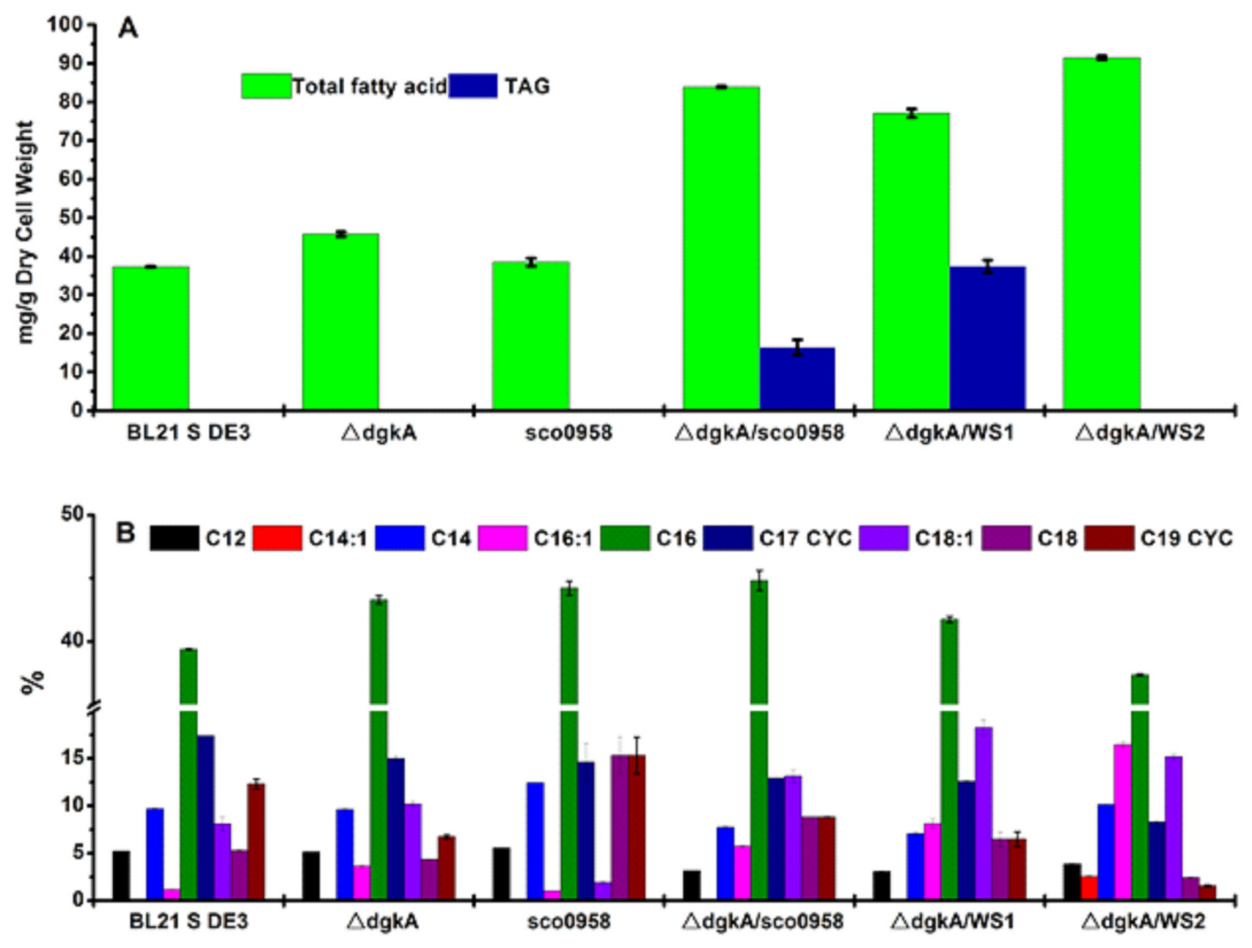
Total amounts of fatty acids and triacylglycerol (A) in strains BL 21 Star^TM^ DE3, △*dgkA*, SCO0958, △*dgkA*/SCO0958, △*dgkA*/WS1, and △*dgkA*/WS2 were measured individually by GC-FID. The fatty acid compositions of these strains were also determined (B). All strains were cultured in LB at 30 °C for 48 hrs. Data presented are averages of two replicate cultures and error bars represent the standard error.

GC-FID quantification showed that the total amount of TAG was 16.4 mg/g dry cell weight and 37.4 mg/g dry cell weight in strain △*dgkA*/SCO0958 and △*dgkA*/WS1, respectively (Figure 5A). To evaluate the effect of the TAG biosynthesis pathway on fatty acid production in *E. coli*, the total amount of fatty acids in strains BL 21 Star^TM^ DE3, △*dgkA*, SCO0958, △*dgkA*/SCO0958, △*dgkA*/WS1, and △*dgkA*/WS2 was measured (Figure 5A). For strain △*dgkA*, the total amount of fatty acids in *E. coli* increased slightly to 45.7 mg/g dry cell weight from 37.3 mg/g dry cell weight in BL21 Star^TM^ DE3. Heterologous expression of SCO0958 alone in BL21 Star^TM^ DE3 did not affect production of fatty acids in *E. coli*. With heterologous expression of enzymes SCO0958, WS1, and WS2 in strain △*dgkA*, the total amount of fatty acids increased 2.25, 2.06, and 2.45 fold to 84, 77, 91 mg/g dry cell weight, respectively, compared to BL21 Star^TM^ DE3. Apparently, introducing the TAG biosynthesis pathway, or the WE biosynthesis pathway in the case of the WS2 enzyme, does improve the total amount of fatty acids in *E. coli*. 

Cellular fatty acid composition was also determined (Figure 5B). In BL21 Star^TM^ DE3, the fraction of cyclopropane fatty acids (C17 CYC and C19 CYC) was 29.7%, while only 9.3% was unsaturated fatty acids (C14:1, C16:1 and C18:1). The cyclopropane fatty acid fraction was reduced to 21.8%, 19.1%, and 9.8% in strains △*dgkA*/SCO0958, △*dgkA*/WS1, and *dgkA*/WS2, respectively; whereas the unsaturated fatty acid fraction was increased to 18.9%, 26.4%, and 34.2%, respectively. The saturated fatty acid fraction (C12, C14, C16, and C18) was not changed significantly by overexpressing WS/DGAT enzymes in strain △*dgkA*. Microbial fuels with a high proportion of unsaturated fatty acids possess favorable low temperature flow properties, improving operation under winter conditions [42]. However, when *E. coli* enters stationary phase from log phase, unsaturated fatty acids are converted to cyclopropane fatty acids due to modification of the *cis* double bond by cyclopropane fatty acid synthase [43,44]. It appeared that the introduction of the TAG or WE biosynthesis pathway reduced the conversion of unsaturated fatty acids to cyclopropane fatty acids. Thus, introduction of the TAG or WE biosynthesis pathway into *E. coli* not only increases the total amount of fatty acids, but also improves the fuel properties of fatty acids.

As described above, strain △*dgkA*/WS1 was more effective in producing TAG than strain △*dgkA*/SCO0958. Hence, enzyme WS1 was used in further TAG production studies, which are presented below. 

**Figure 6:**
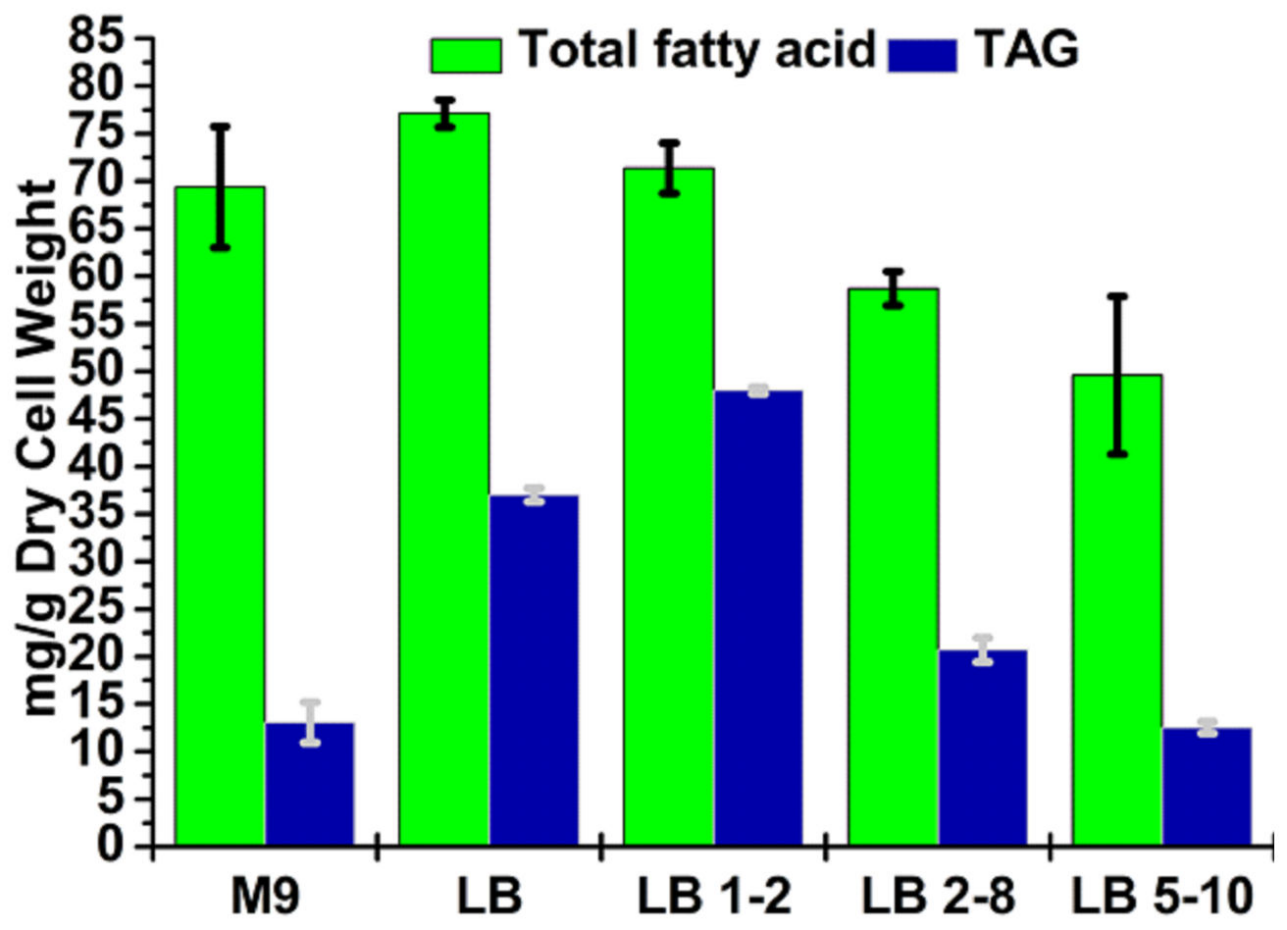
Total amounts of fatty acids and triacylglycerol in strain △*dgkA*/WS1 cultured in the minimal medium M9, rich medium LB, and LB supplemented with different concentrations of glucose and sodium bicarbonate. LB 1-2: LB supplemented with 1% glucose and 2 g/L sodium bicarbonate; LB 2-8: LB supplemented with 2% glucose and 8 g/L sodium bicarbonate; LB 5-10: LB supplemented with 5% glucose and 10 g/L sodium bicarbonate. Data presented are averages of two replicate cultures and error bars represent the standard error.

Next, we tested the effect of media on TAG production. The amounts of TAG and fatty acids produced by strain △*dgkA*/WS1 were compared among five different culture media (Figure 6). These tested culture media included the minimal medium M9, the rich medium LB, and LB supplemented with varied concentrations of glucose and sodium bicarbonate (LB 1-2: 1% glucose and 2 g/L sodium bicarbonate, LB 2-8: 2% glucose and 8 g/L sodium bicarbonate, and LB 5-10: 5% glucose and 10 g/L sodium bicarbonate). As shown in Figure 6, using M9, the most cost-effective medium for industrial operation, only a small amount of TAG, 13 mg/g dry cell weight, was produced. It appears that the minimal medium does not provide a favorable condition for TAG production, as demonstrated here and in a previous study by Santala et al. [45]. The rich medium was more effective and the largest amount of TAG, 49 mg/g dry cell weight, was observed in LB supplemented with 1% glucose and 2 g/L sodium bicarbonate strain. When the concentrations of glucose and sodium bicarbonate were further increased, less TAG and fatty acids were produced. Overall, our results on TAG production represents a significant improvement over previous efforts. For instance, a recent study reported TAG production of 6.7 mg/g dry cell weight in a mutant *Acinetobacter baylyi* ADP1 strain with four gene deletions (*poxB*, *dgkA*, *metY* and a triacylglycerol lipase) in MA/9 minimal medium supplemented with sodium gluconate and glycerol as carbon and energy sources [28].

### Combining modification of central carbon metabolism and triacylglycerol biosynthesis for fatty acid production

**Figure 7:**
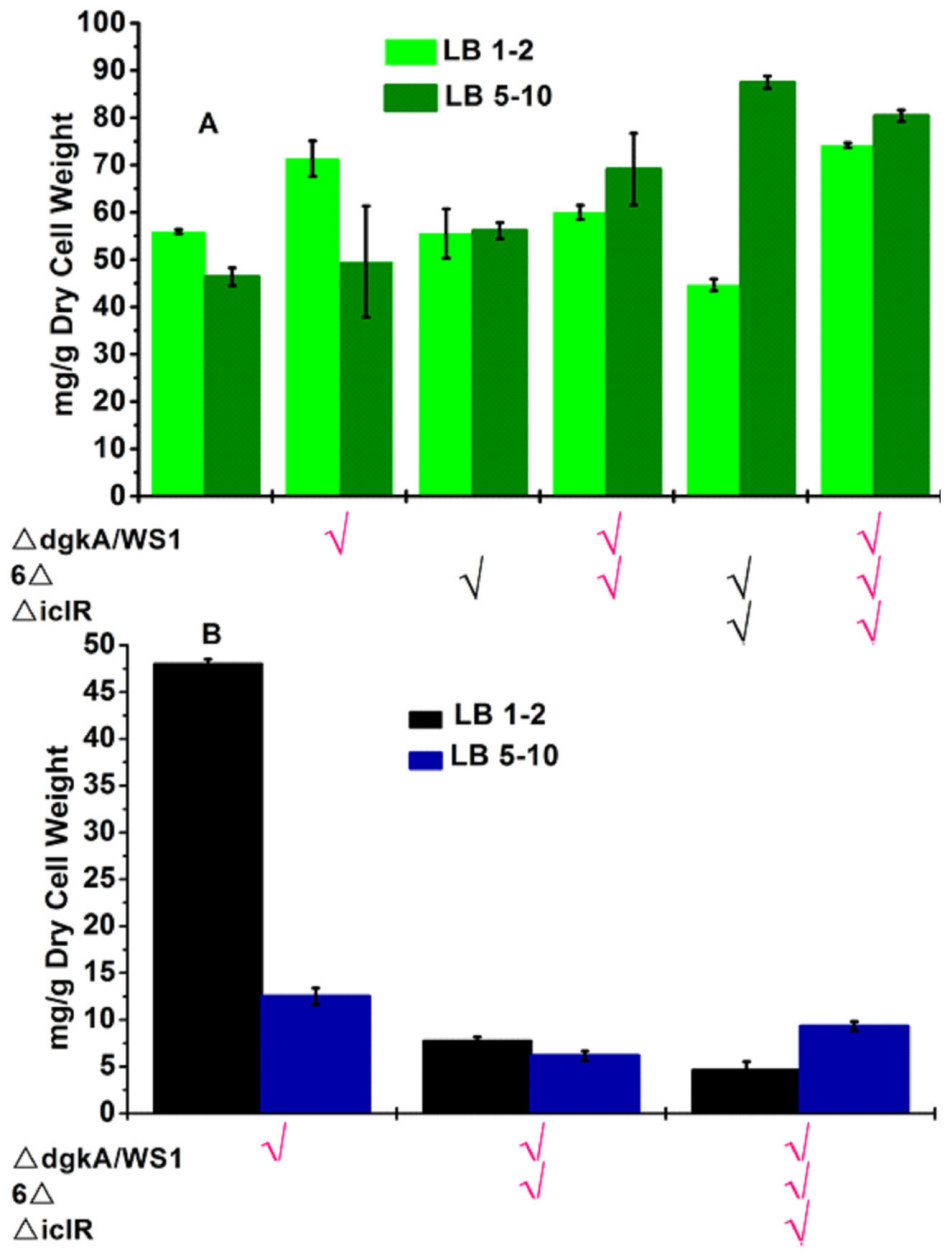
Total amounts of fatty acids (A) and triacylglycerol (B) in strains containing both central carbon metabolism modifications and the triacylglycerol biosynthesis pathway. 6△ refers to six knockouts of △*cyoA*△*adhE*△*nuoA*△*ndh*△*pta*△*dld*; 7△ combines 6△ and △*iclR*. Two culture media, LB 1-2 and LB 5-10, were tested. All strains were cultured at 30 °C for 48 hrs. Data presented are averages of two replicate cultures and error bars represent the standard error.

As reported above, we have improved fatty acid production in *E. coli* by modifying central carbon metabolism. On the other hand, we were able to produce TAG for storing fatty acid carbon chains by introducing a heterlogous TAG biosynthesis pathway. To determine if the combination of these two strategies would further improve fatty acid production, we introduced the TAG biosynthesis pathway into strains △*cyoA*△*adhE*△*nuoA*△*ndh*△*pta*△*dld* and △*cyoA*△*adhE*△*nuoA*△*ndh*△*pta*△*dld*△*iclR* (also termed 6△ and 7△, respectively, hereafter). The culture medium LB 1-2, which favored TAG production in the previous section, was utilized in characterizing the resulting strains. Results of total fatty acids are shown in Figure 7A. Compared to the wild type, the total amount of fatty acids produced was improved in strains △*dgkA*/WS1 and 7△-△*dgkA*/WS1. All other strains showed comparable or even reduced total fatty acid contents. We also measured the fatty acid contents in these strains in another culture medium LB 5-10 with higher concentrations of glucose and sodium bicarbonate (Figure 7A). Interestingly, strain 7△, which had the lowest amount of fatty acids in LB 1-2, was the top performer in LB 5-10, reaching a fatty acid content of 87.5 mg/g dry cell weight. The combined strain 7△-△*dgkA*/WS1 also performed very well, producing the second highest fatty acid content of 80.4 mg/g dry cell weight. During the strain construction process, we also introduced the TAG biosynthesis pathway into another knockout strain ∆*cyoA*∆*adhE*∆*nuoA*, which showed the third largest improvement on fatty acid production (Figure 2). In addition, we explored the deletion of *fadE* to remove the competing β–oxidation pathway. Unfortunately, the amount of TAG was actually reduced in these modified strains (Figure S2). 

We also compared the amounts of TAG in the three strains containing the WS1 enzyme (Figure 7B). In LB 1-2, modifications of the central carbon metabolism caused a significant decrease of TAG production in both strains 6△-△*dgkA*/WS1 and 7△-△*dgkA*/WS1, compared with strain △*dgkA*/WS1. In LB 5-10, the TAG levels in all three strains were low; strain 6△-△*dgkA*/WS1 was again less efficient in producing TAG compared to strain △*dgkA*/WS1, whereas the additional knockout of *iclR* recovered part of the decrease in strain 7△-△*dgkA*/WS1.

The above results indicated that even though each strategy by itself could increase fatty acid production, combining them synergistically is not straightforward. The challenge is essentially due to the complexity of cellular metabolism and regulation in *E. coli*. How the cell responds to a genetic modification, in terms of fatty acids or TAG production, depends on both the culture conditions and the genetic background of the strain. Most notably, the manipulations on central carbon metabolism we investigated in this work were suggested for minimum medium with glucose as the sole carbon source; whereas it was obvious that TAG production using the WS1 enzyme preferred the rich medium. Moreover, introduction of the TAG biosynthesis pathway affects the metabolic/regulatory network of *E. coli*, which was not considered by the computational model used in this study. In fact, when the TAG biosynthesis pathway was added into the model, further exploration suggested that a different set of gene modifications could be optimal for maximizing TAG production, which will be investigated in our future work. Additionally, we observed that as the number of gene deletions increased, the resulting knockout strains became increasingly inefficient in growth (as indicated by the final OD in Figure 2D and growth rates). The severely perturbed cellular environment in strains 6△ and 7△ might negatively affect the expression and function of the WS1 enzyme, causing reduced TAG production. More extensive study of the metabolic and regulatory networks related to fatty acid biosynthesis and a fundamental understanding of the *in vivo* mechanisms of WS/DGAT enzymes will be needed to further advance metabolic engineering of *E. coli* for production of fatty acids and its derivatives for biofuel applications.

## Conclusion

In this work, we aimed to engineer *E. coli* strains for improved fatty acid production. First, we sought to modify the central carbon metabolic and regulatory network to increase flux through the fatty acid biosynthesis pathway. We carried out a series of gene knockouts suggested by a computational model and demonstrated improvement in fatty acid content. Meanwhile, we introduced a TAG biosynthesis pathway into *E. coli*, which not only increased the total amount of fatty acids, but also improved the fuel properties of the fatty acids. Finally, we combined both strategies in *E. coli* with the goal of further increasing fatty acid production. Despite mixed results, our work provides new insights and suggests valuable directions for future investigation. In addition, the recombinant *E. coli* strains generated in this work could serve as bases for further engineering efforts, which might ultimately lead to microbial cell factories capable of producing and accumulating abundant fatty acids for bio-hydrocarbon production. 

## Supporting Information

Figure S1 **The total amount of fatty acids in recombinant *E. coli* strains genetically modified in the order suggested by *in silico* modeling (black columns) and in three other random combinations (grey columns).**
(TIF)

Figure S2 **TAG production in four additional knockout strains: 1-∆*dgkA*/pBAD; 2-∆*fadE*∆*dgkA*/pBAD;3-∆*cyoA*∆*adhE*∆*nuoA*∆*dgkA*/pBAD;4-∆*cyoA*∆*adhE*∆*nuoA*∆*fadE*∆*dgkA*/pBAD.** Cells were cultured in LB and collected at 24 hrs after induction for TAG analysis by GC-FID.
(TIF)
